# Everybody's Page

**Published:** 1890-05-03

**Authors:** 


### Everybody's Page.

"Everything in this world depends on distinctness of idea and firmness of purpose."

The largest number of suicides which has ever been reported in Berlin during a single month occurred in January of the present year. There were 75, of which 10 were children.

Numerous contrivances have at various times been introduced with a view to diminishing the bad effects resulting from nicotine in pipes, and now we hear of yet another invention. The smoke, by passing through cotton wool charged with pyrogallic acid, is said to be freed from nicotine.

People are said to have incurred lead-poisoning from merely biting off or passing through their mouth silk sewing-thread. Silk thread is soaked in acetate of lead to increase its weight, and the danger is quite possible; and apart from the danger the habit of biting off cotton or silk, instead of using scissors, is not the most improving thing for the teeth.

Abernethy was most enthusiastic about his lectures at the hospital, and couldn't bear to miss one of them. One afternoon one of his friends met him looking very smart in a white waistcoat, and said to him, "You are looking very gay today, sir." "Ay," said Abernethy, "one of my girls was married this morning." "Then, sir, you ought to have given yourself a holiday, and not come to lecture to-day." "Holiday, sir, why I came down to lecture the day I was married myself." This was enthusiasm with a vengeance.

Some would-be clean people when they feel a slight deafness or humming in their ears make up their minds that they can relieve it by syringing their ears themselves. For instance, if too much wax is secreted by the small glands in the ear, and it forms a mass, an aurist using a syringe would most likely be able to remove it, while an amateur would send the water right on to the surface of the mass, and thus drive it further in. A favourite instrument with nurses is a hairpin; they wrap it up in a corner of a towel, and ram it into unfortunate little baby ears, and often do irreparable injury.

The "New York Medical Record," a week or two ago, wrote a short article on sanitation on ocean steamers, pointing out that hospitals on ships are often put to other than their intended purposes, so that if needed, they would be unavailable. The "Record" complains, too, that the managers do not provide a satisfactory medical service for the vessels, who should be entrusted with the sanitary control of the vessels, and suggests that during the coming summer, when many physicians will be crossing over to the Berlin International Congress, they shall investigate ship sanitation for themselves.

Anti-pyrin is being protested against in Philadelphia as being anything but a "safe cure" for headaches. Bad symptoms, it is stated, have resulted from the taking of it, and in one case the result was nearly fatal.

M. Jacques Bertillon in a recent work shows that Paris has not been so free from typhoid fever for twenty-four years as she was in 1888, the most satisfactory feature in this improvement being the fact that the diminution during the past six years has been progressive.

Dr. Edward F. Brush, speaking at the New York Academy of Medicine in March last, said he was quite sure that it is an entire mistake to suppose that rural, as compared with urban populations, are free from tuberculosis. He thinks that this view, which is very generally held, is due to the fact that the records of vital statistics are not so carefully kept in the country districts as in the towns, and so the causes of deaths are frequently unknown.

The Abbé Touroude has been waging war with his pen against hypnotism, as practised by ignorant people. He considers the fascination which experiments by this power exercises over people very bad, and says that hypnotism in ignorant hands is injurious to health, morals, and religion. This may be so, but "mystery" is dear to the heart of the public, and as long as there are performances of this kind the audience will not be wanting.

Now that the vast improvement in the state of our workhouses is becoming more apparent every day, and when educated women are so often chosen by wise guardians to fill the post of matron, it is interesting to read what King William III., nearly two centuries ago, said the condition of these places ought to be, "Workhouses under a prudent and good arrangement will answer all the ends of charity to the poor, in regard to their souls and bodies; they may be made, properly speaking, nurseries for religion, virtue, and industry."

Suffocation from escape of gas has been reported several times lately in the papers. Two women died on Saturday last, from whose house the authorities had removed the meter and plugged the service pipe, but the plugging gave way and the two poor things were stifled. A few weeks ago a baby was suffocated from escape of gas from a chandelier which had been pulled down to heat some food. Some people have scarcely any sense of smell, others say cheerfully if an escape is pointed out to them: "Yes, it is nasty; I don't think the joints fit," but disastrous consequences never enter their heads. Gas escapes should be seen to at once, however slight.

